# Irf7 regulates the expression of *Srg3* and ferroptosis axis aggravated sepsis-induced acute lung injury

**DOI:** 10.1186/s11658-023-00495-0

**Published:** 2023-11-09

**Authors:** Xinyu Ling, Shiyou Wei, Dandan Ling, Siqi Cao, Rui Chang, Qiuyun Wang, Zhize Yuan

**Affiliations:** 1 Department of Thoracic Surgery, Shanghai Pulmonary Hospital, School of Medicine, Tongji University, Shanghai, 200433 China; 2 Department of Anesthesiology, Shanghai Pulmonary Hospital, School of Medicine, Tongji University, Shanghai, 200433 China; 3 Department of Anesthesiology, Fudan University Shanghai Cancer Center, Shanghai, 200032 China; 4 School of Clinical Medicine, Weifang Medical University, Weifang, 261053 Shandong China; 5 Medical Department, Shanghai Pulmonary Hospital, School of Medicine, Tongji University, Shanghai, 200433 China; 6 Department of Anesthesiology, Ruijin Hospital, Shanghai Jiaotong University School of Medicine, Shanghai, China

**Keywords:** Sepsis-induced acute lung injury, *Srg3*, Irf7, Ferroptosis, NF-κB signaling pathway

## Abstract

**Objective:**

To investigate the mechanism of action of *Srg3* in acute lung injury caused by sepsis.

**Methods:**

First, a sepsis-induced acute lung injury rat model was established using cecal ligation and puncture (CLP). RNA sequencing (RNA-seq) was used to screen for highly expressed genes in sepsis-induced acute lung injury (ALI), and the results showed that *Srg3* was significantly upregulated. Then, SWI3-related gene 3 (*Srg3*) was knocked down using AAV9 vector in vivo, and changes in ALI symptoms in rats were analyzed. In vitro experiments were conducted by establishing a cell model using lipopolysaccharide (LPS)-induced BEAS-2B cells and coculturing them with phorbol 12-myristate 13-acetate (PMA)-treated THP-1 cells to analyze macrophage polarization. Next, downstream signaling pathways regulated by *Srg3* and transcription factors involved in regulating *Srg3* expression were analyzed using the KEGG database. Finally, gain-of-loss functional validation experiments were performed to analyze the role of downstream signaling pathways regulated by *Srg3* and transcription factors involved in regulating *Srg3* expression in sepsis-induced acute lung injury.

**Results:**

*Srg3* was significantly upregulated in sepsis-induced acute lung injury, and knocking down *Srg3* significantly improved the symptoms of ALI in rats. Furthermore, in vitro experiments showed that knocking down *Srg3* significantly weakened the inhibitory effect of LPS on the viability of BEAS-2B cells and promoted alternative activation phenotype (M2) macrophage polarization. Subsequent experiments showed that *Srg3* can regulate the activation of the NF-κB signaling pathway and promote ferroptosis. Specific activation of the NF-κB signaling pathway or ferroptosis significantly weakened the effect of *Srg3* knockdown. It was then found that *Srg3* can be transcriptionally activated by interferon regulatory factor 7 (Irf7), and specific inhibition of Irf7 significantly improved the symptoms of ALI.

**Conclusions:**

Irf7 transcriptionally activates the expression of *Srg3*, which can promote ferroptosis and activate classical activation phenotype (M1) macrophage polarization by regulating the NF-κB signaling pathway, thereby exacerbating the symptoms of septic lung injury.

**Supplementary Information:**

The online version contains supplementary material available at 10.1186/s11658-023-00495-0.

## Highlights

1. *Srg3* is highly expressed in sepsis-induced acute lung injury.
2. Knockdown of *Srg3* suppresses lung injury symptoms in septic rats.
3. *Srg3* regulates NF-κB signaling pathway and ferroptosis.
4. Irf7 transcriptionally regulates *Srg3* and activates its transcriptional activity in sepsis-induced acute lung injury.
5. The overexpression of Irf7 weakens the beneficial effect of *Srg3* knockdown on improving sepsis-induced acute lung injury.

**Supplementary Information:**

The online version contains supplementary material available at 10.1186/s11658-023-00495-0.

## Introduction

Sepsis is a life-threatening condition and a common syndrome with a high mortality rate, which can affect various organs of the body and cause a systemic inflammatory response [1, 2]. The lungs are the most commonly involved organs in sepsis. Accompanied by the excessive activation of inflammatory mediators, the dysregulation of the body’s response to these inflammatory mediators leads to a positive feedback loop of sustained amplification and destruction, as well as a cytokine storm, which exacerbates the sepsis-induced acute lung injury (ALI) and progresses to acute respiratory distress syndrome (ARDS) [3]. In the initial stage, if timely diagnosis and effective treatment measures are not taken, it can easily develop into more severe respiratory failure, with a mortality rate of up to 70% [4]. The detailed pathogenesis of ALI/ARDS is still unclear, and it involves a complex network of signals composed of numerous molecules and their signal pathways. *Srg3*, also known as Brg/Brm-associated factor 155 *BAF155*), is a gene that encodes for a subunit of the mammalian SWI/SNF chromatin remodeling complex. This complex plays a crucial role in regulating gene expression through its ability to modulate chromatin structure and accessibility [5].

Studies have shown that various immune cells play important regulatory roles in the development of ALI/ARDS. Macrophages are an important type of innate immune cell in the body, with strong phagocytic ability and involvement in important biological processes such as immune defense, immune self-stabilization, and immune surveillance, as well as regulating the occurrence and development of ALI/ARDS [6, 7]. When macrophages are stimulated by different local environmental factors, they exhibit two distinct polarization states: the classical activation phenotype (M1) and the alternative activation phenotype (M2) [8]. M1 is closely related to proinflammatory responses, while M2 plays a key role in antiinflammatory responses [9].

In ALI, M1 macrophages participate in the acute inflammatory response, while M2 macrophages are mainly involved in counteracting inflammation [10]. Recent research has confirmed that the polarization state of macrophages is the key determinant of the resolution phase. Macrophage polarization, pyroptosis, phagocytosis, and vesicles from different cells are involved in the inflammatory process of ALI, and they are able to dynamically transition between the proinflammatory M1 and immunosuppressive M2 polarized phenotypes in response to changes in microenvironmental signals [11]. If M2 macrophages are effectively promoted, it can downregulate the inflammatory response in ALI and facilitate the healing and repair of the injury. Properly mediating the transition between M1 and M2 states can be crucial for the treatment of ALI [12]. Currently, research suggests that targeting the regulation of macrophage polarization is a new and more effective approach to treat and combat ALI. This involves altering the distribution of M1/M2 macrophages in the lungs, which could become a new direction for ALI treatment research. Yafeng liang et al. reported eliminating IL-33 decreased the levels of MMP2 and MMP9 in bronchoalveolar lavage fluid (BALF) and alleviated lung injury in ALI rats [13]. However, the molecular mechanisms of regulating macrophage polarization in lung injury are still not well understood.

In this study, we used CLP induction to establish a sepsis-mediated rat model of lung injury. Through RNA-seq, we identified *Srg3* as a key player in the development of sepsis-induced ALI. Upregulation of *Srg3* in response to sepsis suggests its potential involvement in the pathogenesis of ALI, which can aid in better understanding the molecular processes leading to lung injury during sepsis. Besides, *Srg3* is involved in sepsis-induced lung injury and can promote M1 polarization of macrophages, leading to ferroptosis and worsening symptoms of sepsis-induced acute lung injury.

## Material and methods

### Sequences, primer, antibodies, and ELISA kit

The shRNA targets *Srg3* and *Irf7* are exhibited in Table 1.

**Table 1:** shRNA ds-oligo sequences

Symbol	Sequences(5′–3′)
sh-SRG3-no. 1	CACCGCACAGCCAAGAGCACAAACACGAATGTTTGTGCTCTTGGCTGTGC
sh-SRG3-no. 2	CACCGGTGAATGGGATTTCTTAAGCAACGGCTTAAGAAATCCCATTCACC
sh-IRF7-no. 1	ACCTCGTGACCCTCAACACCCTAATATCAAGAGTATTAGGGTGTTGAGGGTCACTT
sh-IRF7-no. 2	ACCTCGCTGATCCGCACAAGGTGTATTCAAGAGATACACCTTGTGCGGATCAGCTT

The primer for qRT-PCR or ChIP-qPCR is exhibited in Table 2**.**

**Table 2:** qPCR primers

Symbol	Fvd	Rvs
*SRG3*	TCACAACCCCAAGCACAAGT	AACATCTGTCACACGGGGAC
*GPX4*	CTCCATGCACGAATTCGCAG	TATCGGGCATGCAGATCGAC
*COX-2*	TGAGTACCGCAAACGCTTCT	TCTGGACGAGGCTTTTCCAC
*IRF7*	GGGTCAGTGGGCTTGATTCA	AGGAGGCCTAGTGTGTAGGG
*GAPDH*	TGATGGGTGTGAACCACGAG	AGTGATGGCATGGACTGTGG
*SRG3-promoter*	GAGACGGGGTTTCACCATGT	GGCTGTGTCACGGACAGTAA

The antibodies utilized for western blot, immunohistochemistry, and immunofluorescence are exhibited in Table 3.

**Table 3:** Antibodies

Target	Dilution	Catalog	Application
SRG3	1:200 ~ 1:1000	PA5-55,058, ThermoFisher	WB, IHC
GPX4	1:50 ~ 1:1000	GTX54095, Genetex	WB, IHC
COX-2	1:50 ~ 1:1000	GTX60935, Genetex	WB, IHC
IRF7	1:50 ~ 1:1000	MA5-41,165, ThermoFisher	WB, IHC, ChIP
SP-C	1:50	ab90716, Abcam	IHC
iNOS	1:2000	ab283655, Abcam	IHC
Arg1	1:1000	ab259271, Abcam	IHC
CD86	1:1000	13,395–1-AP, Proteintech	FCS
CD206	1:1000	18,704–1-AP, Proteintech	FCS
p65	1:1000	ab16502, Abcam	WB
Phos-p65	1:50 ~ 1:1000	ab76302, Abcam	WB, IHC, IF
Ikbkb	1:1000	ab178870, Abcam	WB
phos-Ikbkb	1:50 ~ 1:1000	ab194528, Abcam	WB, IHC, IF
GAPDH	1:1000	MA1-16,757, ThermoFisher	WB
Goat anti-rabbit IgG H&L (HRP)	1:20,000	ab205718, Abcam	WB, IHC
Donkey anti-rabbit IgG H&L (HRP)	1:20,000	ab205722, Abcam	WB, IHC

The ELISA kit’s detail is exhibited in Table 4.

**Table 4:** ELISA kit

Target	Catalog
Il4	R4000, R and D system
Il5	ab267811, Abcam
Il13	ab269547, Abcam

The detail of commercial merchandise used in the research is exhibited in Table 5.

**Table 5:** Commercial merchandise

Product	Catalog
JSH-23	S7351, Selleck
AMG-232	S1316, Selleck

### Ethics declaration

All animal experiments were approved by the Institutional Animal Care and Use Committee of Shanghai Pulmonary Hospital (approval no. K23-179Y). The animals were housed in a facility with appropriate environmental conditions and received a standard diet and water ad libitum. We took measures to minimize the number of animals used in the study and to reduce their suffering, including the use of anesthesia during surgical procedures. We followed the principles of the three Rs (replacement, reduction, and refinement) in the design and implementation of this study.

### Animal experiment

Forty-eight male Sprague–Dawley rats (6–8 weeks) were purchased from Beijing Vital River Laboratory Animal Technology (Beijing, China). All rats were kept 2 rats per each cage in a specific pathogen-free animal laboratory with a temperature-controlled colony and 12/12 h light/dark cycle with free access to chow and water, and 42 rats were left until 20–21 months. The CLP-induced septic rate model was based on previous description [14]. Briefly, the rats were anesthetized by intraperitoneal injection of ketamine (K) and  xylazine (X) [55 mg/kg (K) + 10 mg/kg (X)]. Half of the cecum-free end was ligated and punctured twice with a 21-gauge needle. The cecum contents were squeezed out, the cecum was repositioned, and an incision was sutured in the muscle and skin layers. Subsequently, the rats were subcutaneously injected with saline (1 mL/100 g) at 37 °C, and returned to the cage to rewarm for 1 h. The control group had an abdominal incision, but their cecum was not ligated and punctured.

### Bronchoalveolar lavage fluid (BALF)

The rat was anesthetized using an appropriate anesthetic agent and the anesthesia was maintained throughout the procedure. The rat was placed in a supine position on a surgical table and the limbs were secured to prevent movement. The skin was opened over the trachea using sterile surgical instruments to expose the trachea. Sterile scissors were used to make a small incision in the trachea and to insert a sterile cannula into the trachea. The lungs were infused with saline solution through the cannula and the fluid was withdrawn back into a sterile syringe. Saline solution was gently injected and aspirated several times to wash the lungs and collect the fluid. The fluid was collected in a sterile tube or container and centrifuged to remove cellular debris. The supernatant should then be stored at −80°C or processed immediately for analysis.

### Wright–Giemsa staining

For each bronchoalveolar lavage fluid (BALF) sample, centrifugation was carried out at 300*g* for 10 min using a cytocentrifuge, utilizing a slide to immobilize the cells. Subsequently, the slide was subjected to staining using a Wright–Giemsa Stain Kit (ab245888, Abcam), following the instructions provided by the manufacturer.

### Histological staining

After euthanizing the rats, the lung tissues were taken out and fixed in neutral formaldehyde solution (10%) for 24 h, and then dehydrated with gradient concentration ethanol (70%, 80%, 90%, 95%, and 100%). After xylene (Solarbio, China) was transparent, the lung tissues were embedded in paraffin and cut into 5-μm sections. The sections were treated with xylene for 4 min, differentiated in ethanol hydrochloric acid for 10 s, rinsed and stained with eosin (Solarbio, China) solution again for 2 min, fixed with neutral gum, and then the lung injury and inflammatory cell infiltration were observed under a DMM-300D microscope (Caikon, China). The experiment was repeated in triplicate.

### PAS staining

PAS (Periodic acid–Schiff) staining is a special staining technique that can detect the presence of complex carbohydrates and other glycoproteins in tissue sections. Generally, after deparaffinizing and rehydrating them using xylene and graded ethanol solutions, subsequently the slides were washed in distilled water for 5 min. The sections were incubated with 0.5–1% periodic acid at room temperature for 5–10 min, followed by a rinsing of the sections with distilled water for 5 min. The sections were incubated with Schiff reagent at room temperature for 15–30 min and kept protected from light. The sections were rinsed with running tap water for 5–10 min. To counterstain the sections, they were incubated with hematoxylin for 1–2 min, followed by a rinsing with tap water for 5–10 min. The sections were dehydrated using graded ethanol solutions, and then cleared in xylene. Finally, the sections were mounted with a suitable mounting medium.

### Immunohistochemical staining

The extracted rat lung tissue was dewaxed until hydration after routine inclusion and then dropped with 3% H_2_O_2_ at room temperature for 15 min, then with normal goat serum blocking solution at room temperature for 15 min. After cleaning, 50 μL iNOS, Arg1, Spc-1, phos-p65, and phos-Ikbkb (Abcam, UK) primary antibody were added and incubated at 4℃ overnight. After that, secondary antibody (1:500, ab199091, Abcam) was added and incubated at 37 ℃ for 15 min, and then 40 μL horseradase-labeled streptomycin working liquid was added and incubated at 37 ℃ for 15 min for DAB color reaction. After rinsing with distilled water, hematoxylin was used for restaining for 30 s, and then the tablets were dehydrated and sealed. Five nonoverlapping visual fields were selected for observation in each section under the microscope, and cells showing brown–yellow or brown–brown particles in the nucleus were positive cells. All experiments were conducted independently three times, and average values were taken for analysis.

### TUNEL assay

Terminal deoxynucleotidyl transferase dUTP nick end labeling (TUNEL) assay is a commonly used technique to detect apoptotic cells in tissue sections. Briefly, 5-µm thick sections were mounted on glass slides. Next, the sections were deparaffinized using xylene or other deparaffinization agents and rehydrated through graded alcohol solutions. The TUNEL reaction mixture was prepared according to the manufacturer’s instructions and applied to the tissue sections, incubating for 1 h at 37 °C in a humidified chamber. After incubation, the sections were washed with PBS buffer to remove unbound TUNEL reagents. The sections were counterstained with 4′,6-diamidino-2-phenylindole (DAPI), and then mounted using mounting medium and coverslip. Finally, the sections were observed under a fluorescence microscope or confocal microscope to detect apoptotic cells.

### Lung permeation determination

Rats were utilized in these experiments and were anesthetized prior to insertion of a catheter into the jugular vein. Subsequently, Evans blue (EB) dye was administered intravenously, and it traveled through the blood vessels to reach the lung tissue. After 1–2 h, the animals were sacrificed, and the lungs were harvested, followed by a saline flush to eliminate any excess dye. The lung tissue was then homogenized in a buffer solution to extract the EB dye that had accumulated in the tissue. Finally, a spectrophotometer was used to measure the amount of EB dye in the tissue, which provided a measure of the lung tissue’s permeability.

### RNA-seq

The lung tissue samples in three groups (*n* = 6 for each) for mRNA-sequencing (mRNA-seq) were collected as described below in the animal experiments section. These lung tissue samples were conducted using a CloudSeq mRNA enrichment kit and Illumina HiSeq sequencer (Thermo Fisher Scientific, Waltham, MA, USA) by Jgenebook Biotech Ltd. (Wuhan, China). Differentially expressed genes (DEGs) between the ALI model rats and sham rats were identified using Cuffdiff software (part of the Cufflinks software). The thresholds of the DEGs were set as fold change (FC) log |FC| ≥ 1.0 and *P* ≤ 0.05 with a fragments per kilobase million (FPKM) value ≥ 0.1, and at least one criterion was expected to be satisfied. Differentially expressed mRNA clustering was performed using FPKM values with the heat map function of R package. The RNA-seq data were deposited in the GEO database (the GEO no. is not available yet owing to the verification).

### Immunofluorescence

To preserve protein structure and prevent degradation, the cells or tissue sample were fixed with paraformaldehyde. The cells or tissue were permeabilized using Triton X-100, and nonspecific binding sites on the sample were blocked with BSA to prevent the primary antibody from binding nonspecifically. Afterward, the sample was incubated with a primary antibody that specifically binds to the protein of interest. The primary antibody bound to the target protein, and any unbound primary antibody were removed through washing. Next, the sample was incubated with a fluorescent dye-conjugated secondary antibody, which bound to the primary antibody. Then, the sample was washed to remove any unbound secondary antibody and mounted on a slide with DAPI. Finally, a fluorescence microscope was used to analyze the sample.

### qRT–PCR

Using the RNeasy Plus Mini kit (Tsingke, China), RNA in the cell was extracted from BEAS-2B cells and tissues. RNA expression was evaluated using the 2^−ΔΔCT^ method, and GAPDH was used as an internal reference for genes.

### Western blotting (WB)

First, the protein amount was quantified to extract proteins from cells or tissues. Next, the protein sample was mixed with a loading buffer, heated, and loaded onto a polyacrylamide gel that contained molecular weight markers. An electric current was applied to the gel to separate the proteins by their molecular weight. Then, the separated proteins were transferred from the gel to the polyvinylidene difluoride (PVDF) membrane. The membrane was incubated with a blocking buffer to prevent nonspecific binding of the primary antibody. Afterward, the membrane was incubated with a primary antibody that specifically recognizes the protein of interest. Subsequently, the membrane was incubated with a secondary antibody that was conjugated with an enzyme, which bound to the primary antibody. A substrate was added to the membrane that reacted with the enzyme conjugated to the secondary antibody, producing a visible signal. Finally, a chemiluminescence imaging system was used to capture and analyze the signal intensity.

### Coculture

THP-1 monocytes (no. GDC0100, CCTCC) were cultured in RPMI 1640 supplemented with 1% penicillin–streptomycin and 5% FBS. To induce THP-1 monocytes to become M0 macrophages, 50 ng/mL phorbol 12-myristate 13-acetate (PMA, Sigma–Aldrich, Shanghai) was added to the cells (10^6^ cells/mL) and incubated for 24 h. THP-1 cells with a concentration of 10^6^ cells/mL were inoculated into the upper chamber of the 24-well Transwell plate, and BEAS-2B (no. GDC0139, CCTCC) containing each group were inoculated into the lower chamber of a Transwell system (10^5^ cells/mL). After coculture for 24 h, cells were collected for subsequent detection.

### CCK-8

BEAS-2B were cultured on 96-well cell culture plates with 5 × 10^3^ cells in each well. After 72 h of culture, CCK-8 solution (Thermofisher, USA) was added to the medium at the ratio of 10 mL/100 mL and incubated at 37℃ for 4 h. Optical density (OD) was measured at 450 nm using an iMark microplate reader (Bio-Rad, USA). Cell viability was determined by calculating absorbance values from reference standard curves. The analysis was repeated in triplicate.

### Flow cytometry

To perform flow cytometric analysis to identify M1 or M2 macrophages, single-cell suspension was prepared from the BALF fluid. Following this, cells were incubated with antibodies that were specific to surface markers associated with M1 (CD86) or M2 (CD206) macrophages. The cells were washed and fixed with formaldehyde, and then permeabilized with a permeabilization buffer. Cells were incubated with FITC-labeled CD86 and PE-labeled CD206 antibodies. Then, the analysis was performed by BD FACS (BD, USA), and gating was done on the macrophage population based on size and granularity. The percentage of M1 or M2 macrophages was determined by analyzing the expression of CD86 or CD206.

### EdU assay

First, cells of interest were plated in a culture dish and maintained under suitable culture conditions. Next, the cells were incubated with a medium containing EdU, which may vary based on the experimental conditions and cell type. Following this, the EdU-containing medium was removed and cells were washed with PBS buffer. It was essential that the cells were fixed using formaldehyde to retain the incorporated EdU. Then, the cells were permeabilized with a suitable permeabilization buffer. After that, a click chemistry reaction mixture was added containing a fluorescent probe to the fixed and permeabilized cells, and incubated for 30 min. Subsequently, the cells were washed with a suitable buffer to eliminate the unreacted click chemistry reagents. Finally, the cells were analyzed using a fluorescence microscope.

### ELISA

The BALF fluid was taken and put into 4 ℃ for 30 min, and then centrifuged at 3000 rpm. An ELISA kit (Abcam, UK) was used to detect the concentration of inflammatory factors in BALF based on rat interleukin 4 (IL-4), IL-5, and IL-13.. A 100 μL standard working solution or sample was added into the corresponding plate hole and incubated at 37 ℃ for 90 min; 100 μL biotinated anti-TNF-α or IL-6 working solution was added and incubated at 37 ℃ for 60 min, and then 100 μL HRP enzyme conjugate working solution was added and incubated at 37 ℃ for 30 min. The 90 μL substrate solution was added and incubated at 37 ℃ for about 15 min. Finally, 50 μL termination solution was added. The iMark microplate reader (Bio-Rad, USA) was used to immediately take readings at a 450 nm wavelength.

### ChIP–qPCR

BEAS-2B cells were directly fixed in the culture medium by adding formaldehyde (HCHO) to achieve a final concentration of 1%. The formaldehyde concentration was derived from a 37% HCHO–10% methanol stock solution (Calbiochem). Following a 20-min incubation with 125 mM glycine to neutralize excess formaldehyde, cells were washed with ice-cold phosphate-buffered saline (PBS) and then lysed in a solution containing 50 mM Tris pH 8.0, 10 mM EDTA, 1% SDS, and protease inhibitors. This mixture was incubated at 4 °C for 30 min. To induce chromatin fragmentation, a COVARIS S220 focused-ultrasonicator was employed, resulting in DNA fragments spanning from 200 to 1000 base pairs. Subsequently, the chromatin was diluted tenfold in a dilution buffer comprising 20 mM Tris pH 8.0, 2 mM EDTA, 1% triton X-100, 150 mM NaCl, and protease inhibitors. Immunoprecipitations were performed with antibody complexes for Irf7 and phos-p65, conducted overnight at 4°C. The resulting immunocomplexes were subjected to multiple washes. This included three washes with buffer TSEI (20 mM Tris pH 8.0, 2 mM EDTA, 1% triton X-100, 150 mM NaCl, 0.1% SDS, and protease inhibitors), followed by three washes with buffer TSEII (20 mM Tris pH 8.0, 2 mM EDTA, 1% triton X-100, 500 mM NaCl, 0.1% SDS, and protease inhibitors). Additionally, a single wash was performed with buffer TSEIII (10 mM Tris pH 8.0, 250 mM LiCl, 1 mM EDTA, 1% NP40, 1% deoxycholate, and protease inhibitors), followed by a final wash with TE pH 8.0. The immunocomplexes were subsequently extracted in TE buffer containing 1% SDS, and protein–DNA cross-links were reversed by overnight heating at 65 °C. DNA was extracted using a PCR purification kit from Qiagen. For each PCR, one-tenth of the immunoprecipitated DNA was utilized, employing primers specific to the promoter region.

### Luciferase reporter assay

Cells at subconfluent levels were transfected using Lipofectamine Plus (Invitrogen) with 100 ng of a *SRG3* or Cox-2-luciferase reporter gene plasmid and 2 ng of the Renilla control reporter pRL-CMV (Promega). After a 48-h period, the cells were subjected with IRF7 expression vector or phos-p65 specific antagonists maslinic acid. To evaluate the promoter activity level, the firefly luciferase activity was quantified relative to the Renilla luciferase activity utilizing the Dual Luciferase Assay System (Promega), in accordance with the manufacturer’s instructions.

### Statistical analysis

SPSS22.0 (IBM SPSS Statistics, USA) was used for statistical analysis. Measurement data were expressed as mean ± SD, and unpaired *t* test was used for comparison between the two groups. One-way ANOVA or two-way ANOVA and Tukey’s postmortem test were used for data comparison among multiple groups. Log rank test was used for post-statistical analysis, with *P* < 0.05 indicating statistically significant differences.

## Results

### *Srg3* is highly expressed in sepsis-induced lung injury

**Fig. 1:**
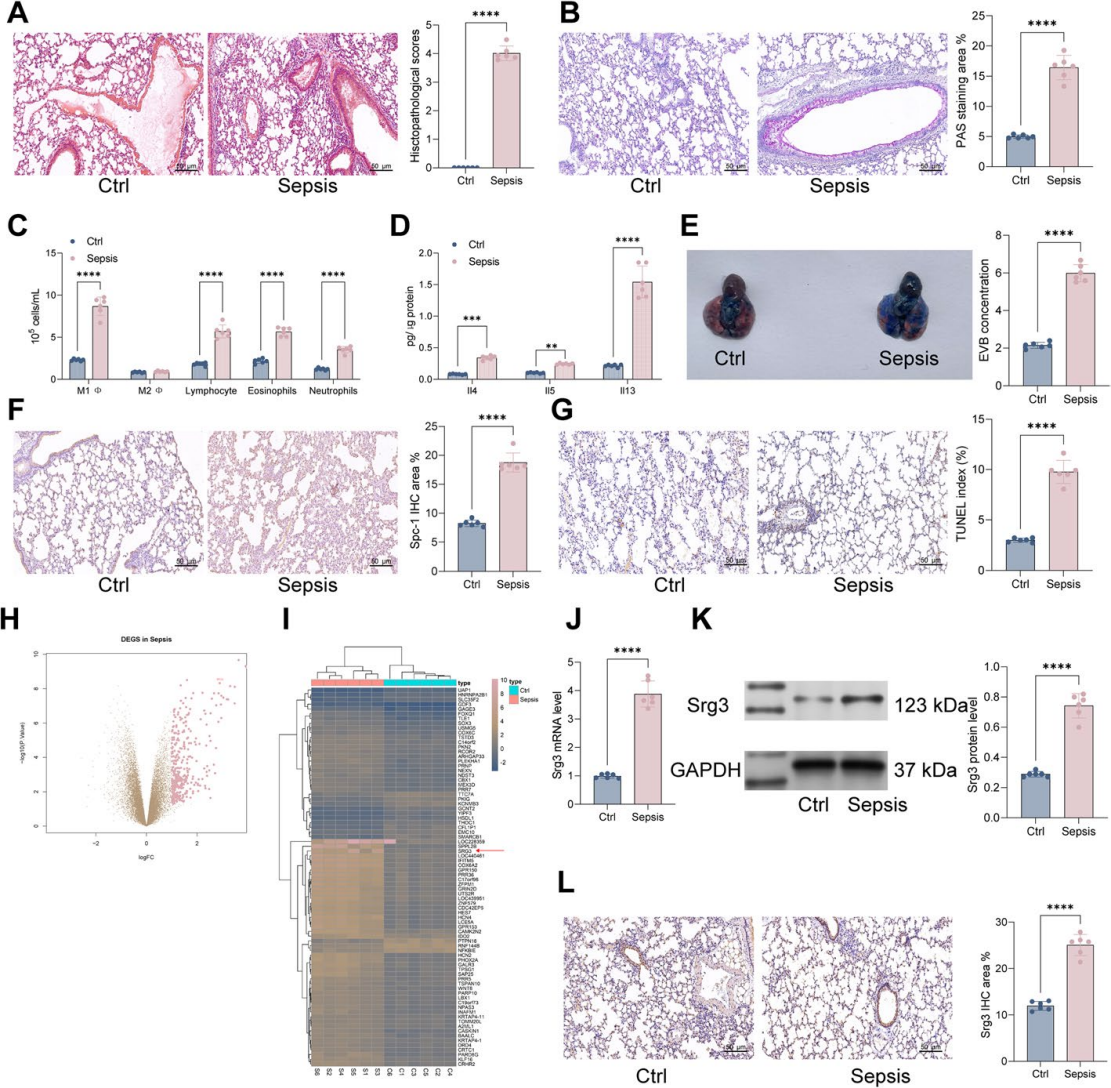
*Srg3* is highly expressed in sepsis-induced lung injury. **A** Histopathology of lung tissues from rats examined by H&E staining. **B** Proportions of glycogen in lung tissues of rats examined by PAS staining. **C** Numbers of various immune cells in bronchoalveolar lavage fluid (BALF) of rats analyzed by flow cytometry. **D** Levels of inflammatory factors in BALF of rats detected by ELISA. **E** Permeability of blood vessels in lung tissues of rats examined by EB staining. **F** Intensity of Spc-1 staining in lung tissues of rats examined by IHC staining. **G** Proportions of apoptotic cells in lung tissues of rats examined by TUNEL staining. **H**, **I** Differential gene expression analysis of lung tissues between control and sepsis-induced rats using RNA-seq. **J**, **K** mRNA and protein expression levels of *Srg3* in lung tissues of rats detected by qPCR and WB, respectively. **L** Intensity of *Srg3* staining in lung tissues of rats examined by IHC staining. Each group contained six rats, with each point representing one rat. Significant differences were detected using one-way ANOVA and Tukey’s multiple comparison test, **P < 0.01, ***P < 0.001, ***P < 0.0001

We first established a septic rat model using the cecal ligation and puncture (CLP) surgery. Pathological observations of lung tissue were performed by hematoxylin and eosin (H&E) and PAS staining. In the sham group, the staining showed intact bronchial and alveolar structures, normal alveolar septa, and no infiltration. In the septic rat group induced by CLP, the H&E staining showed obvious destruction of the bronchial and alveolar structures, thickening of alveolar septa, and increased exudation in the lung interstitium. The PAS staining showed increased mucin and glycol protein, severe airway cell damage, and significant thickening of the tracheal wall and epithelial mucosa (Fig. 1A, B). Furthermore, analysis of the ratio of various inflammatory cells in the bronchoalveolar lavage fluid (BALF) of rats showed a significant increase in the number of neutrophils, eosinophils, lymphocytes, and M1 macrophages in the septic rat group (Fig. 1C). ELISA detection of the inflammatory cytokines IL-4, IL-5, and IL-13 in BALF also showed a significant increase (Fig. 1D).

Lung permeation refers to the ability of a substance to pass through the lung tissue and enter the bloodstream. This process is dependent on the permeability of the lung tissue, which is affected by various factors, such as the size of the substance, its solubility, and the integrity of the lung tissue barriers. The measurement of lung permeation is important in assessing the efficacy and safety of drugs that are intended to be delivered through the lungs, as well as in understanding the pathophysiology of lung diseases that affect the permeability of the lung tissue. Therefore, we used Evans blue staining to analyze the permeability of lung tissue and observed a significant increase in permeability in the septic rat group (Fig. 1E). Subsequently, we used immunohistochemistry (IHC) to detect the Spc-1 staining intensity in the lung tissue of rats and observed a significant increase in Spc-1 staining intensity in the septic rat group (Fig. 1F). The TUNEL staining results showed a significant increase in the proportion of apoptotic cells in the lung tissue of septic rats (Fig. 1G).

To further confirm the genes that play a role in septic lung injury, we used RNA-seq to analyze the differential gene expression in the lung tissue of sham and sepsis rats. We screened a total of 671 genes with differential expression, including 219 upregulated genes and 452 significantly downregulated genes (Fig. 1H, I), among which the expression level of *Srg3* was most significantly increased. Therefore, we used qRT–PCR, western blotting, and IHC to detect the mRNA and protein expression levels of *Srg3* in the lung tissue of rats, and the results were consistent with the RNA-seq results, showing that *Srg3* was significantly upregulated in the lung tissue of septic rats (Fig. 1J–L).

### Knockdown of *Srg3* suppresses lung injury symptoms in septic rats

**Fig. 2:**
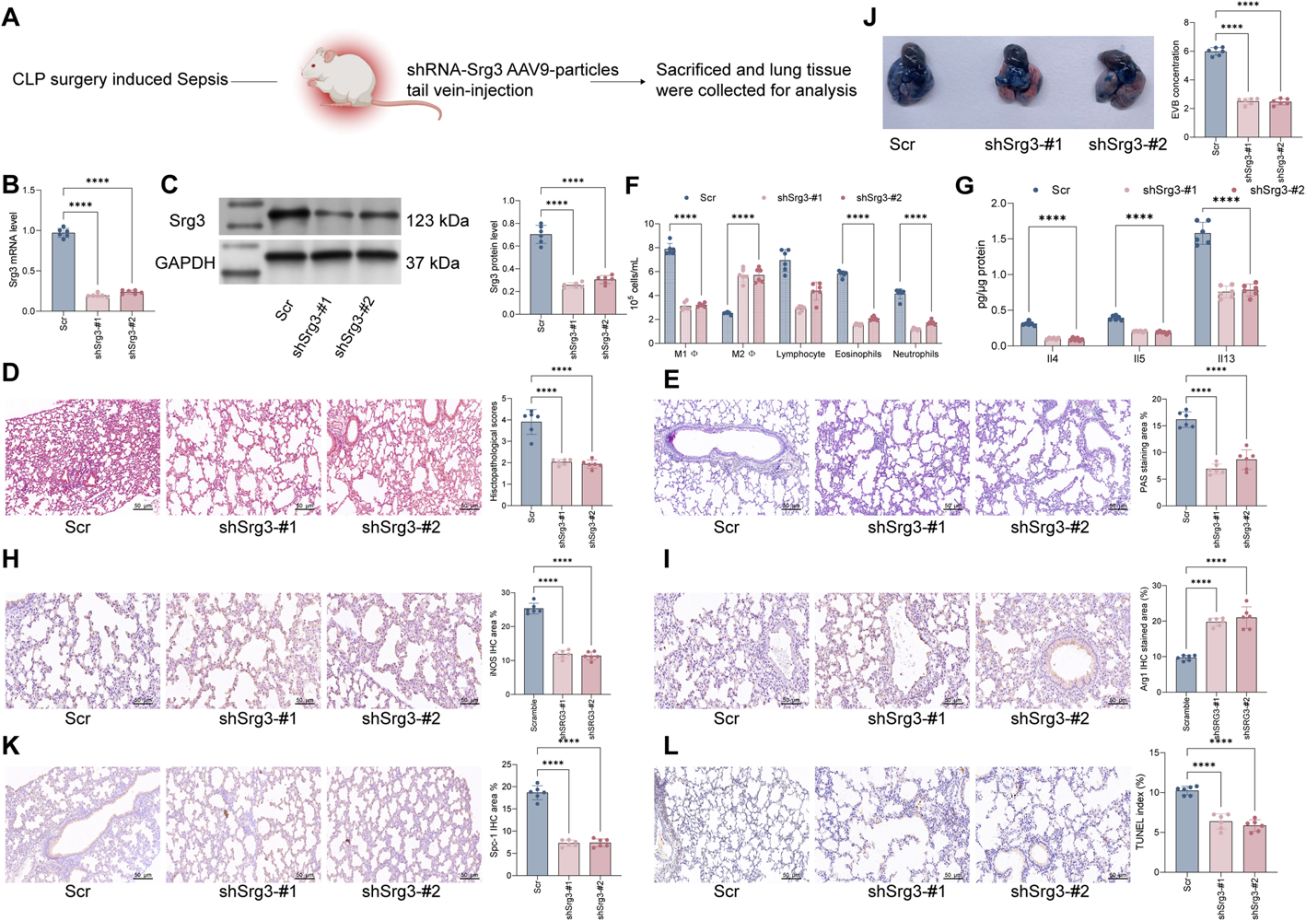
Knockdown of *Srg3* suppresses lung injury symptoms in septic rats. **A** Schematic diagram of injecting the constructed AAV9–shRNA vector targeting *Srg3* into rats via the tail vein. **B**, **C** qRT–PCR and WB detection of *Srg3* mRNA and protein expression levels in rat lung tissue. **D** H&E staining to detect pathological changes in rat lung tissue. **E** PAS staining to observe the proportion of glycogen in rat lung tissue. **F** Flow cytometry analysis of the number of various immune cells in rat BALF. **G** ELISA detection of inflammatory factors in rat BALF. **H**, **I** IHC staining to observe the staining intensity of iNOS and Arg1 in rat lung tissue. **J** EB staining to observe vascular permeability in rat lung tissue. **K** IHC staining to observe the staining intensity of Spc-1 in rat lung tissue. **L** TUNEL staining to detect the proportion of apoptotic cells in lung tissue. Each group contained six rats, with each point representing one rat. Significant differences were detected using one-way ANOVA and Tukey’s multiple comparison test, **P < 0.01, ***P < 0.001, ***P < 0.0001

To further confirm the role of *Srg3* in sepsis-induced lung injury, we constructed an AAV9 viral vector targeting *Srg3* shRNA and injected it into rats via the tail vein (Fig. 2A). First, we used qRT–PCR and WB to validate the knockdown efficiency of shRNA (Fig. 2B, C). The results showed that after knocking down *Srg3*, the pathological changes in rat lung tissue were significantly improved, and the bronchial and alveolar structures were significantly restored, the thickness of alveolar septa was reduced, and interstitial exudation was reduced (Fig. 2D). Moreover, PAS staining showed that after inhibiting the expression of *Srg3*, the number of goblet cells and mucus secretion in rat lung tissue decreased, airway cells were repaired, and the thickness of the tracheal wall and epithelial mucosa was significantly reduced (Fig. 2E). We further analyzed the immunological cells and inflammatory factors in rat BALF, and the results showed that under the action of shSrg3, the number of proinflammatory cells in BALF was significantly reduced, while the content of M2 macrophages increased significantly, accompanied by a decrease in the content of inflammatory factors (Fig. 2F–G). To further confirm that *Srg3* can reduce the number of M1 macrophages and promote the number of M2 macrophages in lung tissue, we used IHC to detect the staining intensity of M1 macrophage marker iNOS and M2 macrophage marker Arg1, and the results showed that after knocking down *Srg3*, the staining intensity of iNOS in lung tissue was significantly reduced, while the staining intensity of Arg1 increased significantly (Fig. 2H, I). Furthermore, we found that after reducing the expression of *Srg3*, the permeability of rat lung tissue was significantly reduced, and the staining intensity of Spc-1 protein and the number of apoptotic cells were significantly reduced (Fig. 2J–L). These results preliminarily indicate that inhibiting the expression of *Srg3* can significantly improve sepsis-induced lung injury.

### *Srg3* knockdown inhibited lipopolysaccharide (LPS)-induced BEAS-2B cell death

**Fig. 3:**
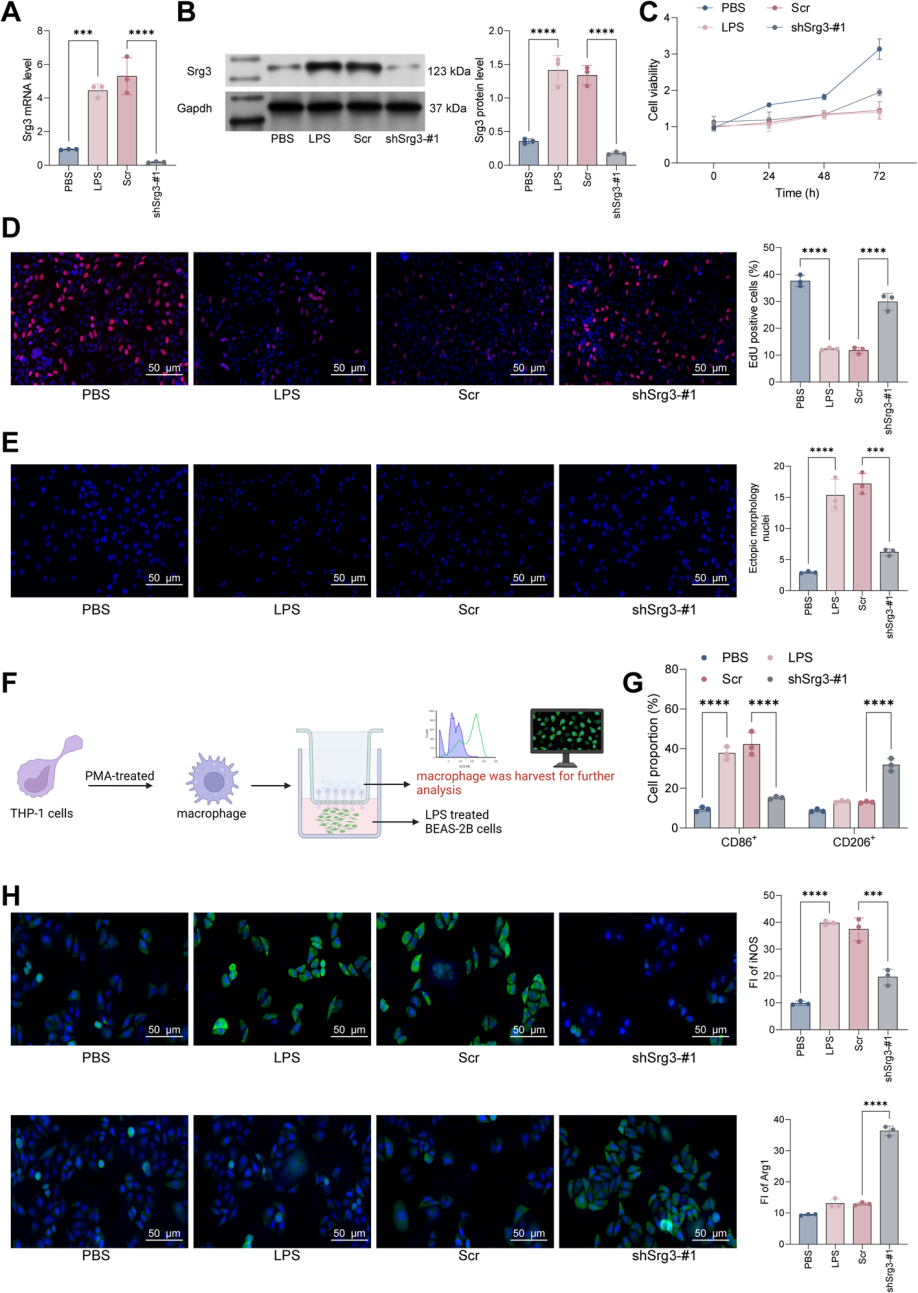
*Srg3* knockdown inhibited LPS-induced BEAS-2B cell death. **A**, **B** qRT–PCR and western blot analysis were performed to detect the mRNA and protein expression levels of *Srg3* in BEAS-2B cells. **C**, **D** CCK-8 and EdU staining were used to analyze the growth activity of BEAS-2B cells. **E** DAPI staining was used to analyze the nuclear morphology of BEAS-2B cells. **F** BEAS-2B and PMA-treated THP-1 cells were cocultured in a Transwell system, and THP-1 cells were collected for subsequent experiments. **G** Flow cytometry was used to detect the proportion of CD86 and CD206 in THP-1 cells after coculture. **H** Immunofluorescence staining was used to detect the fluorescence intensity of iNOS and Arg1 in THP-1 cells after coculture. Each experiment was repeated three times, and the data were presented in the form of mean plus or minus standard deviation. One-way ANOVA or two-way ANOVA was used for significance analysis between the data. After ANOVA, Tukey’s multiple comparison test was used for post hoc test. **P < 0.01, ***P < 0.001, ***P < 0.0001

To further confirm that *Srg3* can promote septic lung injury by affecting the activity of lung epithelial cells, we first used LPS to treat BEAS-2B cells to establish a septic lung injury cell model, and then we knocked down *Srg3* in BEAS-2B cells and verified the knockdown efficiency by qRT–PCR and WB (Fig. 3A, B). Subsequently, we first analyzed cell growth activity using CCK-8 and EdU staining. Results showed that after LPS treatment, the activity of BEAS-2B cells was significantly limited, but after further knocking down *Srg3*, the activity of the cells had a certain degree of recovery (Fig. 3C, D). Additionally, DAPI staining results showed that after LPS treatment, the morphology of BEAS-2B cell nuclei was significantly altered, and the number of abnormal nuclei increased significantly. However, after further knocking down *Srg3*, the number of abnormal nuclei decreased (Fig. 3E). In in vivo experiments, we found that *Srg3* can promote lung injury symptoms by regulating M1 macrophage polarization. Therefore, we cocultured BEAS-2B cells and PMA-treated THP-1 cells in a Transwell system, and then used flow cytometry and immunofluorescence to detect the expression levels of macrophage markers in THP-1 cells. The results showed that after coculturing with LPS-treated BEAS-2B cells, the proportion of M1-polarized THP-1 cells increased significantly, and the staining intensity of iNOS also increased significantly. However, after coculturing with BEAS-2B cells with low *Srg3* expression, the proportion of M2-polarized THP-1 cells increased significantly, and the fluorescence intensity of Arg1 also increased significantly (Fig. 3F–H). Combined with the in vivo experimental results, it can be concluded that *Srg3* can aggravate septic lung injury symptoms by promoting M1 macrophage polarization and inflammatory reactions.

### *Srg3* regulated NF-κB signaling pathway and ferroptosis

**Fig. 4:**
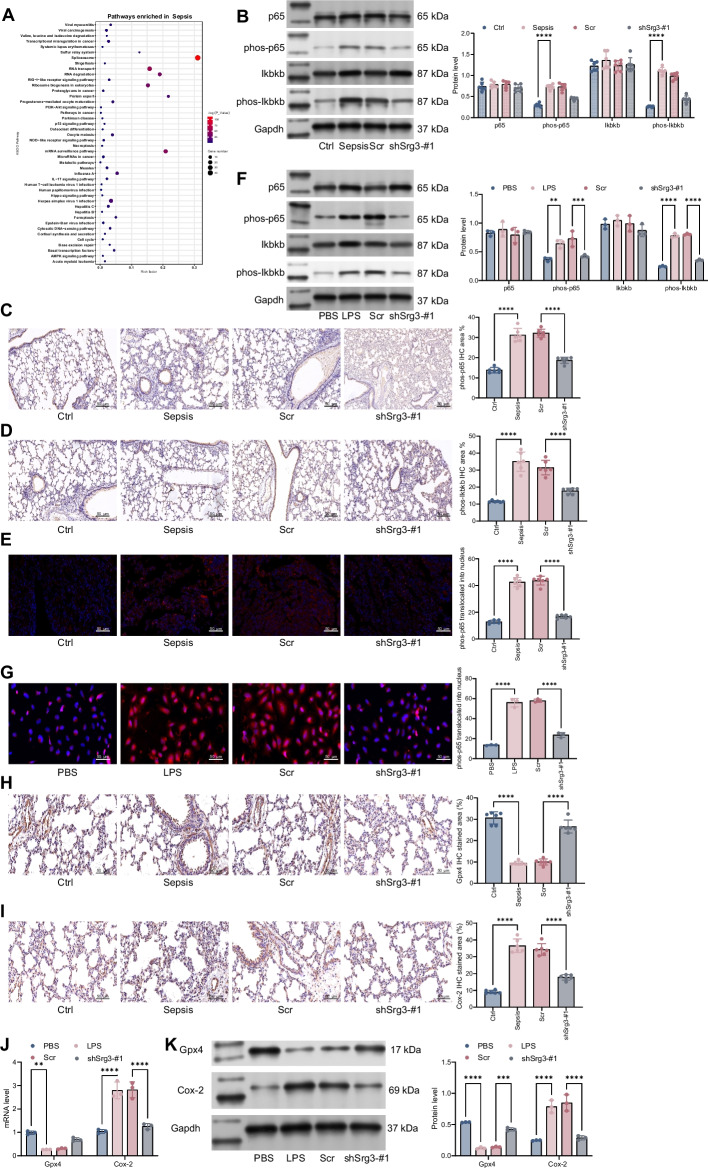
*Srg3*-regulated NF-κB signaling pathway and ferroptosis. **A** We performed KEGG enrichment analysis to identify signaling pathways associated with *Srg3*-regulated genes. **B** Protein expression levels of p65, phos-p65, Ikbkb, and phos-Ikbkb were measured in rat lung tissue. **C**, **D** Immunohistochemistry was used to determine the staining intensity of phos-p65 and phos-Ikbkb in rat lung tissue. **E** Immunofluorescence was used to determine the nuclear localization of phos-p65 in rat lung tissue. **F** Protein expression levels of p65, phos-p65, Ikbkb, and phos-Ikbkb were measured in BEAS-2B cells by WB. **G** Immunofluorescence was used to determine the nuclear localization of phos-p65 in BEAS-2B cells. **H**, **I** Immunohistochemistry was used to determine the staining intensity of Gpx4 and Cox-2 in rat lung tissue. **J**, **K** qRT–PCR and WB were used to measure mRNA and protein expression levels of Gpx4 and Cox-2 in BEAS-2B cells. Each experiment was repeated three times, and the data were presented in the form of mean plus or minus standard deviation. Each group contained six rats, with each point representing one rat. One-way ANOVA or two-way ANOVA was used for significance analysis between the data. After ANOVA, Tukey’s multiple comparison test was used for post hoc test. **P < 0.01, ***P < 0.001, ***P < 0.0001

To further clarify the downstream signaling pathways regulated by *Srg3*, we used KEGG enrichment analysis to identify the signaling pathways enriched by genes related to *Srg3*. From the results, the IL-17 signaling pathway was found to be enriched, which contains several subpathways including MAPK, NF-κB, and AMPK (Fig. 4A). In our preliminary experiments, the activity of the AMPK and MAPK signaling pathways did not show significant changes, while the activity of the NF-κB signaling pathway was significantly increased in sepsis. Further inhibition of *Srg3* expression led to a significant reduction in NF-κB activity. Therefore, we used western blotting to detect the activation of the NF-κB signaling pathway in rat lung tissue. The results showed that after CLP induction, the levels of phos-p65 and phos-IKBKB were significantly increased, but their levels were significantly decreased after further knockdown of *Srg3* (Fig. 4B). Immunohistochemistry showed the same experimental results (Fig. 4C, D). Similarly, by using immunofluorescence analysis to measure the nuclear translocation of phos-p65, we found that the nuclear translocation of phos-p65 was significantly increased in septic rat lung tissue, but it was significantly reduced after further knockdown of *Srg3* expression (Fig. 4E). Similar experimental results were observed in LPS-treated BEAS-2B cells (Fig. 4F-G). Previous studies have shown that DHA-induced ferroptosis of macrophages can cause DNA damage and activate downstream NF-κB, leading to the polarization of macrophages toward an M1 phenotype [15]. Therefore, we used IHC to detect the staining intensity of the ferroptosis-related markers Gpx4 and Cox-2. We observed that the staining intensity of Gpx4 was significantly decreased in septic rat lung tissue, while the staining intensity of Cox-2 was significantly increased. However, after further knockdown of *Srg3*, the staining intensity of Gpx4 in lung tissue was significantly increased, while the staining intensity of Cox-2 was significantly decreased (Fig. 4H, I). Similarly, qRT–PCR and western blotting were used to detect the expression levels of Gpx4 and Cox-2 in BEAS-2B cells. The results showed that after LPS treatment, the expression level of Gpx4 was significantly decreased, while the expression level of Cox-2 was significantly increased. However, after further knockdown of *Srg3*, the expression level of Gpx4 was significantly increased, while the expression level of Cox-2 was significantly decreased (Fig. 4J, K). Besides, specific inhibition of NF-κB signaling pathway by JSH-23 significantly alleviated septic acute lung injury in rats (Additional file 1: Fig. S1). In our experiments, we found that *Srg3* can activate downstream ferroptosis by promoting nuclear translocation of phos-p65. Previous literature reports have indicated that p65, as a transcription factor, can promote the expression of downstream ferroptosis markers such as SLC family proteins and GPX4, thereby promoting ferroptosis. To further validate our findings in lung injury research, we conducted ChIP experiments using anti-phos-p65 antibodies and included the results in the manuscript. We observed that anti-phos-p65 antibodies can enrich more Cox-2 promoter segments. Additionally, we constructed a pGL3 luciferase reporter vector containing the Cox2 promoter sequence and used the p65-specific inhibitor maslinic acid (which inhibits DNA binding activity of NF-κB p65) to show that luciferase activity was significantly reduced in cells treated with MA. These results indicate that *Srg3* can regulate downstream Cox-2 expression by promoting p65 nuclear translocation, thereby activating cellular ferroptosis and exacerbating lung injury caused by sepsis (Additional file 1: Fig. S2A–C). These results fully demonstrate that *Srg3* can promote ferroptosis and activate M1 macrophage polarization by regulating the NF-κB signaling pathway, thereby exacerbating the symptoms of septic lung injury.

### Phorbol esters and erastin treatment weakened shSrg3 function on septic lung injury

**Fig. 5:**
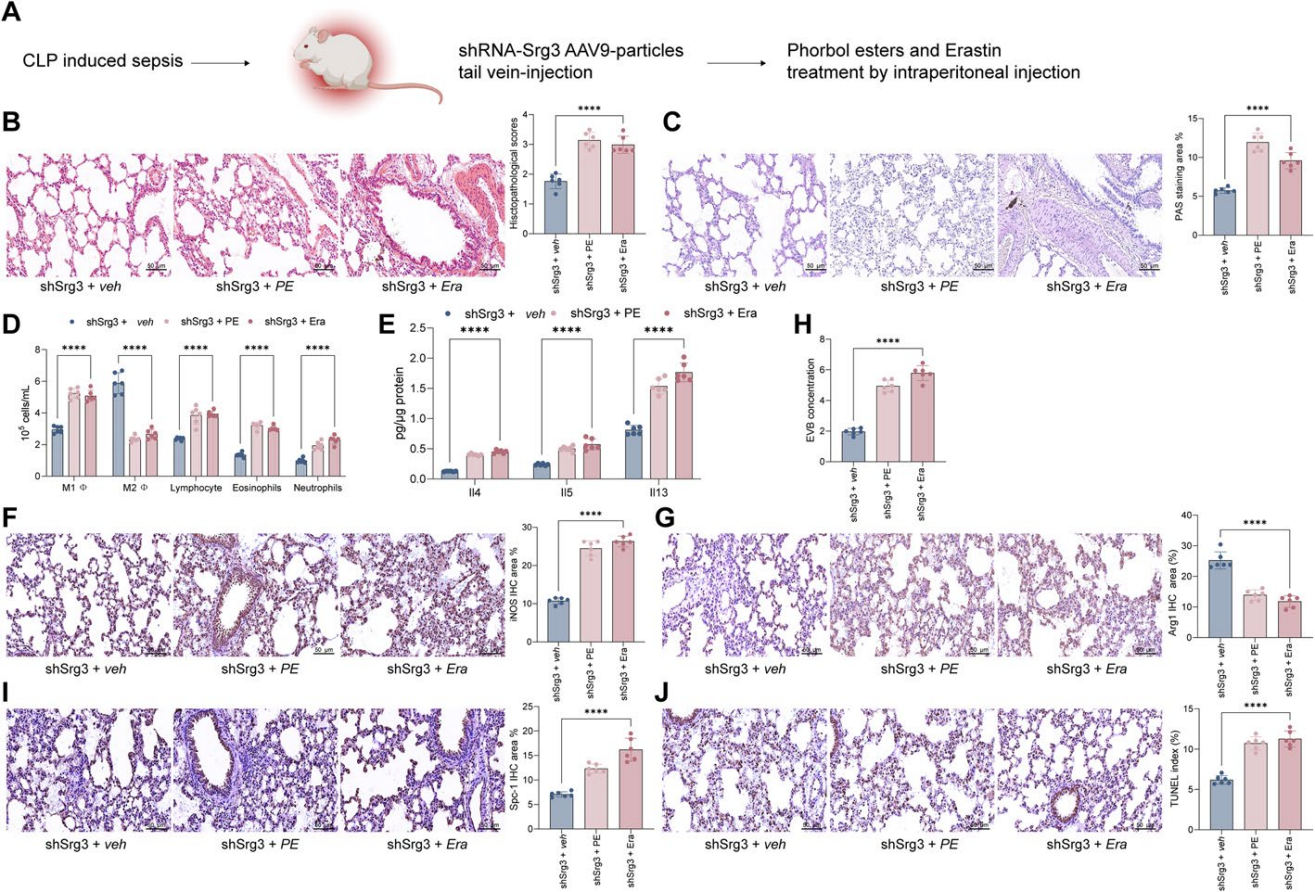
Phorbol esters and erastin treatment weakened shSrg3 function on septic lung injury. **A** Schematic diagram of injecting the constructed AAV9–shRNA vector targeting *Srg3* into rats via the tail vein, then phorbol esters and erastin treatment. **B** H&E staining to detect pathological changes in rat lung tissue. **C** PAS staining to observe the proportion of glycogen in rat lung tissue. **D** Flow cytometry analysis of the number of various immune cells in rat BALF. **E** ELISA detection of inflammatory factors in rat BALF. **F**, **G** IHC staining to observe the staining intensity of iNOS and Arg1 in rat lung tissue. **H** EB staining to observe vascular permeability in rat lung tissue. **I** IHC staining to observe the staining intensity of Spc-1 in rat lung tissue. **J** TUNEL staining to detect the proportion of apoptotic cells in lung tissue. Each group contained six rats, with each point representing one rat. Significant differences were detected using one-way ANOVA and Tukey’s multiple comparison test, **P < 0.01, ***P < 0.001, ***P < 0.0001

To further validate whether *Srg3* can affect acute lung injury caused by sepsis through the NF-κB signaling pathway and ferroptosis, we administered NF-κB activator phorbol esters (PE, as 12-O-tetradecanoylphorbol-13-acetate, 100 μg/kg, intraperitoneal injection) or ferroptosis activator erastin (Era, 40 mg/kg, intraperitoneal injection) to shSrg3-treated rats (Fig. 5A). After treatment with PE or Era, the lung tissue damage in rats was significantly aggravated, with obvious destruction of bronchial and alveolar structures, thickening of alveolar septa, increased leakage in the lung interstitium, increased goblet cell and mucus secretion, severe damage to airway cells, and significant thickening of the tracheal wall and epithelial mucosa (Fig. 5B, C). Further experiments showed a significant increase in proinflammatory cell content and cytokine levels in BALF (Fig. 5D, E). Consistent with our hypothesis, further activation of NF-κB or ferroptosis in rats with knocked-down *Srg3* significantly increased M1 macrophage polarization and decreased M2 macrophage polarization in lung tissue (Fig. 5F, G), accompanied by increased lung tissue permeability and increased apoptotic cell ratio (Fig. 5H, J).

### Irf7 transcriptionally regulates *Srg3* and activates its transcriptional activity in sepsis-induced acute lung injury

**Fig. 6:**
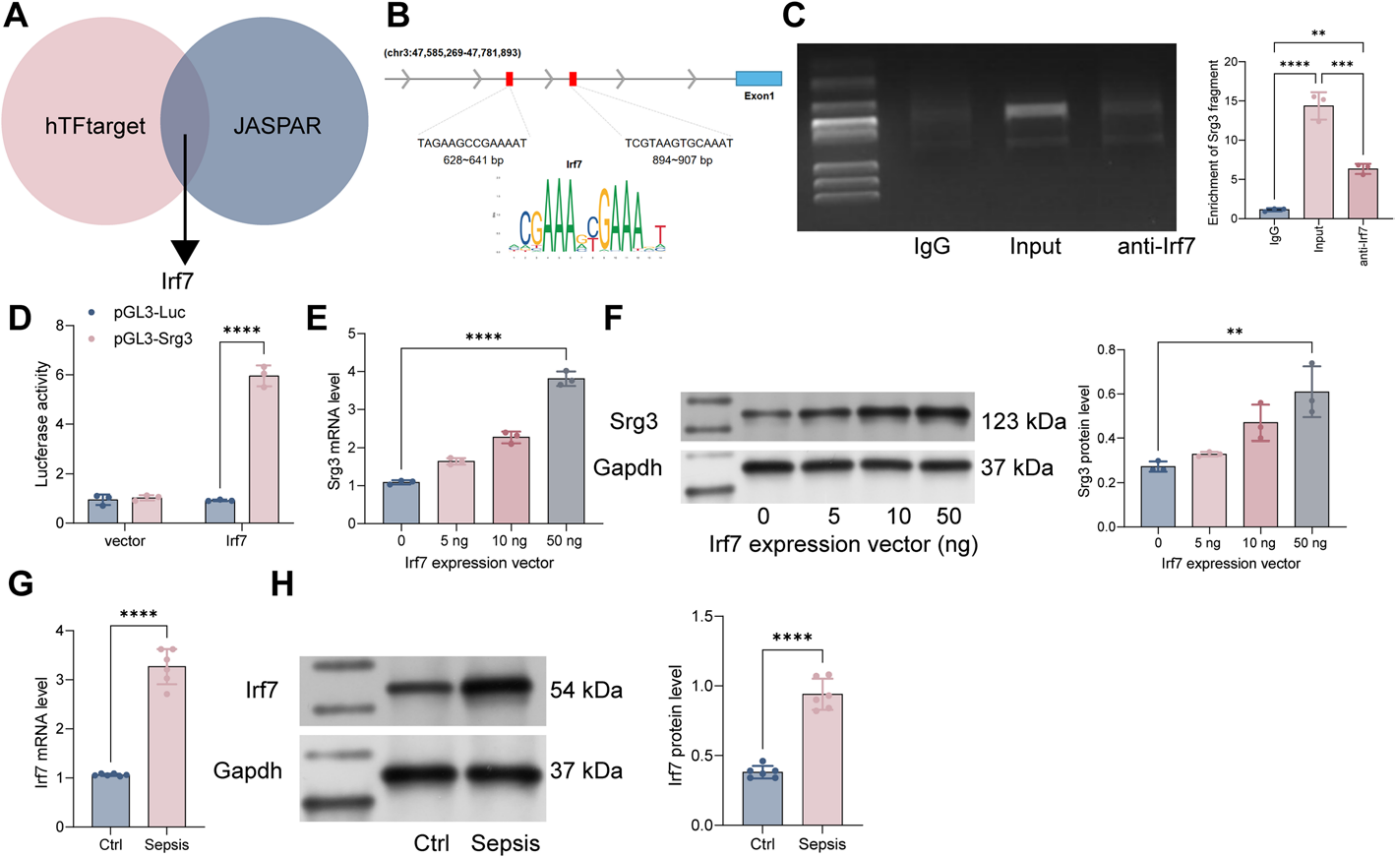
Irf7 transcriptionally regulates *Srg3* and activates its transcriptional activity in sepsis-induced acute lung injury. **A** Prediction of transcription factors that can bind to the *Srg3* promoter using hTFtarget and JASPAR websites. **B** Identification of the conserved binding sequence of Irf7. **C** Verification of the binding relationship between Irf7 and the *Srg3* promoter using ChIP–qPCR experiments. **D** Construction of a luciferase reporter vector containing the *Srg3* promoter sequence and transfection into HEK293T cells along with an overexpression vector of Irf7 to measure luciferase activity. **E**, **F** Measurement of *Srg3* mRNA and protein expression levels in BEAS-2B cells after overexpression of Irf7. **G**, **H** Measurement of Irf7 mRNA and protein expression levels in rat lung tissue using qRT–PCR and WB, respectively. Each experiment was repeated three times, and the data were presented in the form of mean plus or minus standard deviation. Each group contained six rats, with each point representing one rat. One-way ANOVA or two-way ANOVA was used for significance analysis between the data. After ANOVA, Tukey’s multiple comparison test was used for post hoc test. **P < 0.01, ***P < 0.001, ***P < 0.0001

The reason for the high expression of *Srg3* in sepsis-induced acute lung injury has not been clarified. Therefore, we analyzed the transcription factors that can bind to the* Srg3* promoter using the hTFtarget and JASPAR websites and found that Irf7 can bind to the *Srg3* promoter (Fig. 6A, B). Subsequently, we performed ChIP experiments using anti-Irf7 antibody and detected that anti-Irf7 can enrich the *Srg3* promoter fragment (Fig. 6C). We then constructed a pGL3-Luc reporter vector containing the *Srg3* promoter sequence and a mutation vector, which were cotransfected with vector and Irf7 overexpression vector into HEK293T cells. We observed that in cells cotransfected with pGL3-Luc-wt and Irf7, the luciferase activity was significantly increased (Fig. 6D). Moreover, after further overexpression of Irf7 in BEAS-2B cells, the mRNA and protein expression levels of *Srg3* were significantly increased (Fig. 6E, F). These results indicate that Irf7 can transcriptionally regulate *Srg3* expression. Furthermore, we found that Irf7 is significantly upregulated in the lung tissue of septic rats (Fig. 6G, H). Additionally, after treatment with an Irf7-specific inhibitor, the symptoms of acute lung injury in septic rats were significantly attenuated (Additional file 1: Fig S1).

### The overexpression of *Irf7* weakens the beneficial effect of *Srg3* knockdown on improving sepsis-induced acute lung injury

**Fig. 7:**
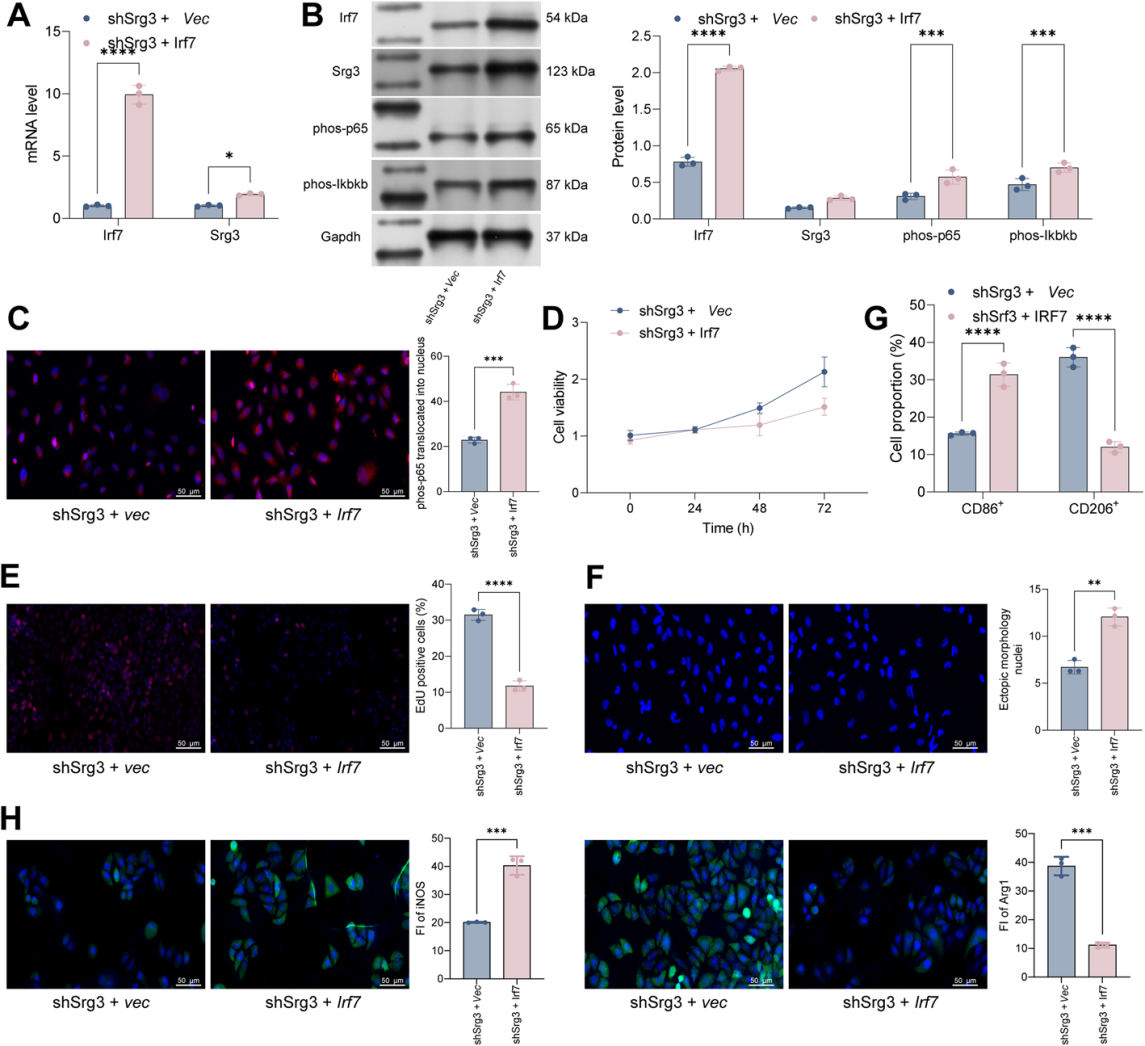
The overexpression of Irf7 weakens the beneficial effect of *Srg3* knockdown on improving sepsis-induced acute lung injury. **A** qRT–PCR analysis was performed to detect the mRNA expression levels of *Srg3* and *Irf7* in BEAS-2B cells. **B** WB analysis was performed to detect the protein level of Srg3, Irf7, phos-p65, and phos-Ikbkb. **C** Immunofluorescence was used to determine the nuclear localization of phos-p65 in BEAS-2B cells. **D**, **E** CCK-8 and EdU staining were used to analyze the growth activity of BEAS-2B cells. **F** DAPI staining was used to analyze the nuclear morphology of BEAS-2B cells. **G** Flow cytometry was used to detect the proportion of CD86 and CD206 in THP-1 cells after coculture. **H** Immunofluorescence staining was used to detect the fluorescence intensity of iNOS and Arg1 in THP-1 cells after coculture. Each experiment was repeated three times, and the data were presented in the form of mean plus or minus standard deviation. One-way ANOVA or two-way ANOVA was used for significance analysis between the data. After ANOVA, Tukey’s multiple comparison test was used for post hoc test. **P < 0.01, ***P < 0.001, ***P < 0.0001

**Fig. 8:**
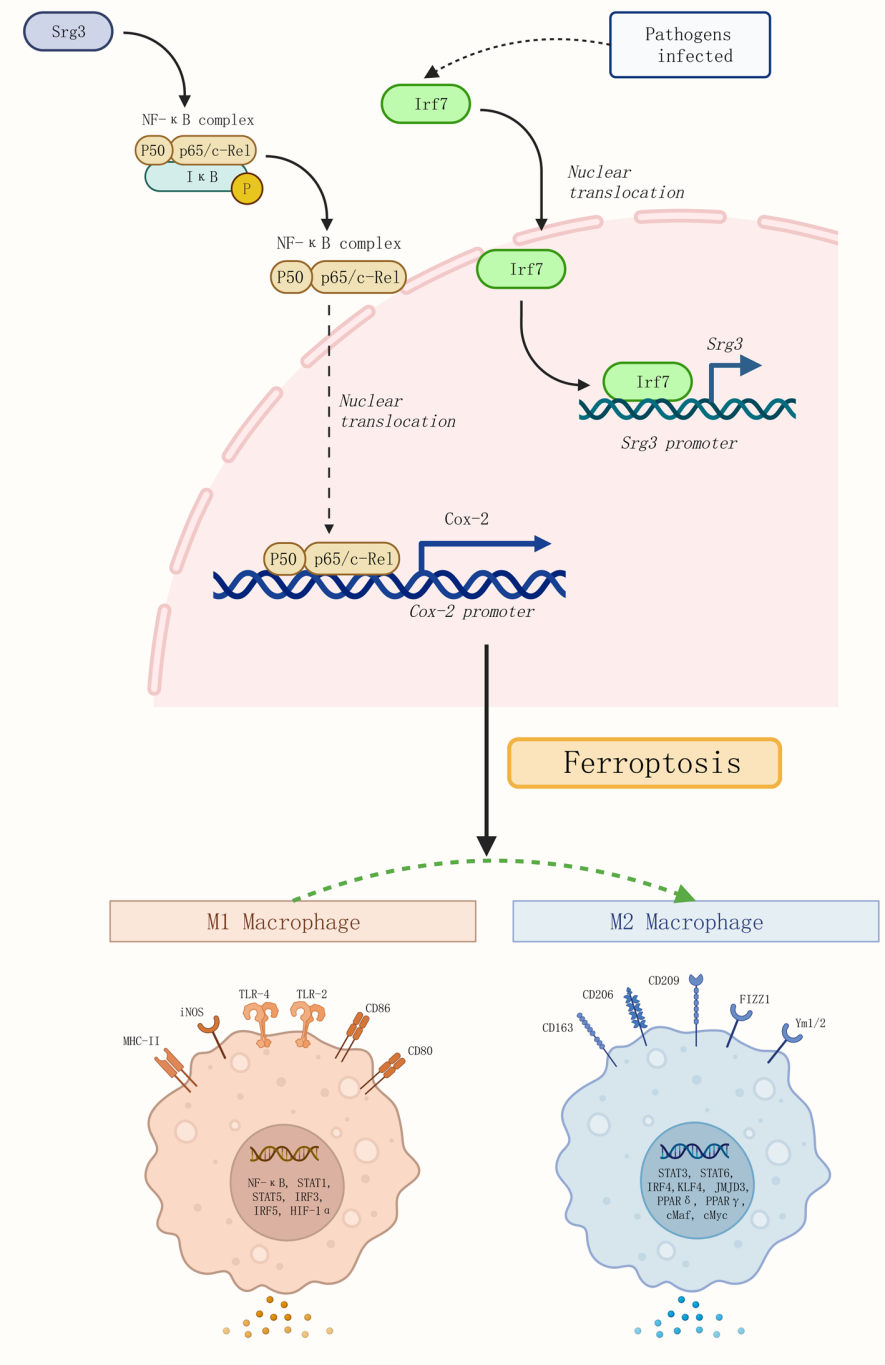
Mechanism illustration. Irf7 can activate the expression of *Srg3* by transcription, thereby increasing the activity of the NF-κB signaling pathway, activating ferroptosis in lung epithelial cells, promoting M1 macrophage polarization, and exacerbating the symptoms of acute lung injury in sepsis

In previous experiments, we confirmed that Irf7 can activate the expression of *Srg3* by transcription, and that inhibiting Irf7 can significantly improve lung injury in rats with sepsis. However, whether Irf7 can participate in macrophage polarization by regulating *Srg3* expression and thereby exacerbating acute lung injury still needs further experimental research. To this end, we further overexpressed *Irf7* in *Srg3*-knockdown BEAS-2B cells and found that the expression level of *Srg3* was significantly increased, and the activity of the NF-κB signaling pathway was increased (Fig. 7A–C). Through CCK-8 and EdU staining, we found that after overexpressing *Irf7*, the decrease in cell viability induced by LPS was significantly exacerbated, and the number of abnormally shaped nuclei was also significantly increased (Fig. 7D–F). Subsequently, after coculturing BEAS-2B cells with PMA-treated THP-1 cells, we found that M1 macrophage polarization of THP-1 cells was significantly increased, and M2 macrophage polarization was significantly decreased (Fig. 7G, H). In summary, Irf7 can activate the expression of *Srg3* by transcription, thereby increasing the activity of the NF-κB signaling pathway, activating ferroptosis in lung epithelial cells, promoting M1 macrophage polarization, and exacerbating the symptoms of acute lung injury in sepsis (Fig. 8).

## Discussion

Inflammation is an important part of the immune response that protects the body from pathogens, but excessive inflammation can cause tissue and organ damage [16]. More and more evidence shows that a variety of pathological changes in lung tissue occur at the onset of ALI. These changes can be increased by capillary permeability, extensive neutrophil infiltration, release of inflammatory mediators, and pulmonary edema, among others [17].

In our study, we first found that the expression of *Srg3* was significantly increased in ALI rats. Furthermore, specific knockdown of *Srg3* in septic rats significantly alleviated the symptoms of ALI caused by CLP surgery. Furthermore, in vitro experiments revealed that knockdown of *Srg3* in BEAS-2B cells significantly alleviated LPS-mediated cell damage. In coculture systems, it significantly inhibited M1 polarization of macrophages, thereby suppressing inflammatory responses. Srg3, also known as Brg/Brm-associated factor 155 (BAF155), is a gene that encodes for a subunit of the mammalian SWI/SNF chromatin remodeling complex. This complex plays a crucial role in regulating gene expression through its ability to modulate chromatin structure and accessibility [5]. Previous studies have implicated *Srg3* in a variety of cellular processes, including embryonic development, stem cell differentiation, and carcinogenesis [18–20]. Furthermore,* Srg3* has been shown to interact with various transcription factors, including c-Myc and NF-κB, highlighting its importance in the regulation of gene expression [21, 22]. Recent research has also suggested a potential role for* Srg3* in the pathogenesis of sepsis-induced lung injury. In a study by Zhang et al., *Srg3* expression was found to be significantly upregulated in rat models of sepsis-induced lung injury [23]. Macrophages, a type of immune cell, have been shown to play a crucial role in lung injury by mediating inflammation and tissue repair [24, 25]. Recent studies have demonstrated that macrophages can also modulate ferroptosis in lung injury [26, 27]. For instance, macrophages can promote ferroptosis by releasing iron or inhibiting ferroptosis through the production of antioxidants or by regulating lipid metabolism [27].

Subsequently, we performed KEGG enrichment analysis and found that genes related to *Srg3* were mainly enriched in the NF-κB signaling pathway. We further observed that the activation of NF-κB signaling pathway, as well as downstream ferroptosis markers, was significantly inhibited in rats and BEAS-2B cells with *Srg3* knockdown. The NF-κB signaling pathway plays a crucial role in regulating the inflammatory response in lung injury. In Sushan Yang’s research, they fathomed 5-methoxyflavone-induced AMPKα activation inhibits NF-κB and P38 MAPK signaling to attenuate influenza A virus-mediated inflammation and lung injury [28], which indicated that blocking NF-κB signaling pathway might be a therapeutic target for sepsis-induced ALI. It is a key transcriptional regulator of various proinflammatory genes that promote the recruitment of inflammatory cells and the release of cytokines and chemokines. Activation of the NF-κB pathway is associated with the pathogenesis of various lung diseases, including ALI/ARDS and pneumonia. Several studies have shown that inhibition of the NF-κB pathway can alleviate the inflammatory response and ameliorate lung injury [29–31]. Ferroptosis is a form of programmed cell death characterized by the accumulation of iron-dependent lipid peroxides, leading to membrane damage and cell death. Emerging evidence suggests that ferroptosis plays a critical role in the pathogenesis of lung injury [32, 33].

To further investigate the mechanism of the upregulation of *Srg3* in ALI, we found that Irf7 could bind to the *Srg3* promoter and promote its transcriptional activation, thereby increasing the expression level of *Srg3*. In subsequent experiments, it was found that the symptoms of ALI in rats were significantly alleviated after treatment with Irf7-specific inhibitor or NF-κB signaling pathway-specific inhibitor. Moreover, overexpression of Irf7 in cells increased the expression of *Srg3* and aggravated the inflammatory response of cells after LPS treatment.

Irf7 is a transcription factor that plays an essential role in the regulation of innate and adaptive immune responses. It is known to induce the production of type I interferon in response to viral infection and other immune stimuli. In addition to its antiviral activity, Irf7 has also been implicated in the regulation of inflammatory processes. Several studies have shown that Irf7 is involved in the regulation of inflammation in various diseases, including autoimmune diseases, viral infections, and cancer. For example, it has been reported that Irf7 deficiency exacerbates autoimmune encephalomyelitis by promoting Th1 and Th17 responses [34]. In a study of hepatitis C virus infection, Irf7 was found to inhibit hepatic inflammation by suppressing the production of proinflammatory cytokines [35]. Additionally, Irf7 has been shown to play a role in the regulation of inflammation in lung cancer by inhibiting the expression of proinflammatory genes [36]. Sepsis-induced ALI is a life-threatening condition often encountered in critical care settings. Our research contributes to the understanding of the molecular mechanisms at play in this condition, potentially paving the way for the development of novel treatment strategies that could significantly impact patient outcomes. In summary, our research sheds light on the role of *Srg3*, NF-κB signaling, and ferroptosis in sepsis-induced ALI, identifies potential therapeutic targets, and provides valuable mechanistic insights. These findings have the potential to advance the field of critical care medicine and guide the development of innovative interventions for septic patients with ALI.

## Conclusions

We fathomed that Irf7 regulates the expression of *Srg3* and transitionally activates the expression of *Srg3*, then activating NF-κB signaling pathway and ferroptosis. Knockdown of *Irf7* or *Srg3* significantly alleviated the symptoms of sepsis-induced lung injury and reduced M1 macrophage polarization in rat lung tissue. Our study may provide insights for the identification of novel therapeutic targets for sepsis and contribute to the development of individualized therapies targeting unique septic lung injury.

### Supplementary Information

**Additional File 1: Fig. S1.** Specific inhibition of the NF-κB signaling pathway or IRF7 weakens sepsis-induced lung injury. **A** Sepsis rats were treated with the NF-κB specific inhibitor JSH-23 or the IRF7 specific inhibitor AMG-232 via gavage. **B**, **C** qRT–PCR was used to detect changes in the expression levels of *SRG3* in lung tissues of rats treated with JSH-23 or AMG-232. **D** H&E staining was used to examine the pathological structure of lung tissues in rats. **E** PAS staining was used to observe the proportion of glycogen in lung tissues of rats. **F** Flow cytometry was used to analyze the number of immune cells in the bronchoalveolar lavage fluid (BALF) of rats. **G** ELISA was used to detect the levels of inflammatory factors in the BALF of rats. **H** EB staining was used to observe vascular permeability in lung tissues of rats. **I** IHC staining was used to observe the staining intensity of SPC-1 in lung tissues of rats. **J** TUNEL staining was used to detect the proportion of apoptotic cells in lung tissues. Each experiment was repeated three times, and the data were presented in the form of mean plus or minus standard deviation. One-way ANOVA or two-way ANOVA was used for significance analysis between the data. After ANOVA, Tukey’s multiple comparison test was used for post hoc test. **P < 0.01, ***P < 0.001, ***P < 0.0001. **Fig. S2.** phos-p65 transcriptional activated Cox-2. **A** Verification of the binding relationship between phos-p65 and the Cox-2 promoter using ChIP–qPCR experiments. **B**, **C** Construction of a luciferase reporter vector containing the Cox-2 promoter sequence and transfection into HEK293T cells along with an DMSO or phos-p65 antagonists maslinic acid to measure luciferase activity. **P < 0.01, ***P < 0.001, ***P < 0.0001.

## Data Availability

The RNA-seq data have been submitted to GEO database. The GEO Accession number will be addressed till the manuscript published.

## Abbreviations

CLP: Cecal ligation and puncture
ALI: Sepsis-induced acute lung injury
*Srg3*: SWI3-related gene 3
LPS: Lipopolysaccharide
PMA: Phorbol 12-myristate 13-acetate
Irf7: Interferon regulatory factor 7
ARDS: Acute respiratory distress syndrome
PAS: Periodic acid–Schiff
TUNEL: Terminal deoxynucleotidyl transferase dUTP nick end labeling
DAPI: 4′,6-Diamidino-2-phenylindole
OD: Optical density
EB: Evans blue
IL-4: Interleukin 4
BAF155: Brg/Brm-associated factor 155

## References

[1] Fleischmann-Struzek C, Mellhammar L, Rose N, Cassini A, Rudd KE, Schlattmann P (2020). Incidence and mortality of hospital- and ICU-treated sepsis: Results from an updated and expanded systematic review and meta-analysis. Intensive Care Med.

[2] Gotts JE, Matthay MA (2016). Sepsis: Pathophysiology and clinical management. BMJ (Clinical research ed).

[3] De Freitas CN, Gaudet A, Portier L, Tsicopoulos A, Mathieu D, Lassalle P (2018). Endocan, sepsis, pneumonia, and acute respiratory distress syndrome. Critical Care (London, England).

[4] Zoulikha M, Xiao Q, Boafo GF, Sallam MA, Chen Z, He W (2022). Pulmonary delivery of siRNA against acute lung injury/acute respiratory distress syndrome. Acta Pharm Sin B.

[5] Wang W (2003). The SWI/SNF family of ATP-dependent chromatin remodelers: Similar mechanisms for diverse functions. Curr Top Microbiol Immunol.

[6] Rubenfeld GD, Caldwell E, Peabody E, Weaver J, Martin DP, Neff M (2005). Incidence and outcomes of acute lung injury. N Engl J Med.

[7] Smith TD, Nagalla RR, Chen EY, Liu WF (2017). Harnessing macrophage plasticity for tissue regeneration. Adv Drug Deliv Rev.

[8] Sica A, Mantovani A (2012). Macrophage plasticity and polarization: in vivo veritas. The Journal of Clinical Investigation.

[9] DeNardo DG, Ruffell B (2019). Macrophages as regulators of tumour immunity and immunotherapy. Nat Rev Immunol.

[10] Gharavi AT, Hanjani NA, Movahed E, Doroudian M (2022). The role of macrophage subtypes and exosomes in immunomodulation. Cell Mol Biol Lett.

[11] Butt Y, Kurdowska A, Allen TC (2016). Acute lung injury: A clinical and molecular review. Arch Pathol Lab Med.

[12] Mokra D, Kosutova P (2015). Biomarkers in acute lung injury. Respir Physiol Neurobiol.

[13] Liang Y, Yang N, Pan G, Jin B, Wang S, Ji W (2018). Elevated IL-33 promotes expression of MMP2 and MMP9 via activating STAT3 in alveolar macrophages during LPS-induced acute lung injury. Cell Mol Biol Lett.

[14] Rittirsch D, Huber-Lang MS, Flierl MA, Ward PA (2009). Immunodesign of experimental sepsis by cecal ligation and puncture. Nat Protoc.

[15] Li LG, Peng XC, Yu TT, Xu HZ, Han N, Yang XX (2022). Dihydroartemisinin remodels macrophage into an M1 phenotype via ferroptosis-mediated DNA damage. Front Pharmacol.

[16] Serhan CN, Chiang N, Van Dyke TE (2008). Resolving inflammation: Dual anti-inflammatory and pro-resolution lipid mediators. Nat Rev Immunol.

[17] Lv H, Liu Q, Wen Z, Feng H, Deng X, Ci X (2017). Xanthohumol ameliorates lipopolysaccharide (LPS)-induced acute lung injury via induction of AMPK/GSK3β-Nrf2 signal axis. Redox Biol.

[18] Lessard J, Wu JI, Ranish JA, Wan M, Winslow MM, Staahl BT (2007). An essential switch in subunit composition of a chromatin remodeling complex during neural development. Neuron.

[19] Han D, Jeon S, Sohn DH, Lee C, Ahn S, Kim WK (2008). SRG3, a core component of mouse SWI/SNF complex, is essential for extra-embryonic vascular development. Dev Biol.

[20] Lessard JA, Crabtree GR (2010). Chromatin regulatory mechanisms in pluripotency. Annu Rev Cell Dev Biol.

[21] Li SG, Shi QW, Yuan LY, Qin LP, Wang Y, Miao YQ (2018). C-Myc-dependent repression of two oncogenic miRNA clusters contributes to triptolide-induced cell death in hepatocellular carcinoma cells. J Exp Clin Cancer Res.

[22] Tanaka T, Maekawa N, Kashio T, Izawa K, Ishiba R, Shirakura K (2017). Tumor necrosis factor α induces the expression of the endothelial cell-specific receptor roundabout4 through the nuclear factor-κB pathway. Biol Pharm Bull.

[23] Jeon S, Seong RH (2016). Anteroposterior limb skeletal patterning requires the bifunctional action of SWI/SNF chromatin remodeling complex in hedgehog pathway. PLoS Genet.

[24] Kolaczkowska E, Kubes P (2013). Neutrophil recruitment and function in health and inflammation. Nat Rev Immunol.

[25] Stockwell BR, FriedmannAngeli JP, Bayir H, Bush AI, Conrad M, Dixon SJ (2017). Ferroptosis: A regulated cell death nexus linking metabolism, redox biology, and disease. Cell.

[26] Gong D, Chen M, Wang Y, Shi J, Hou Y (2022). Role of ferroptosis on tumor progression and immunotherapy. Cell Death Discov.

[27] Dai E, Han L, Liu J, Xie Y, Kroemer G, Klionsky DJ (2020). Autophagy-dependent ferroptosis drives tumor-associated macrophage polarization via release and uptake of oncogenic KRAS protein. Autophagy.

[28] Yang S, Wang L, Pan X, Liang Y, Zhang Y, Li J (2022). 5-Methoxyflavone-induced AMPKα activation inhibits NF-κB and P38 MAPK signaling to attenuate influenza A virus-mediated inflammation and lung injury in vitro and in vivo. Cell Mol Biol Lett.

[29] Brachman RA, McGowan JC, Perusini JN, Lim SC, Pham TH, Faye C (2016). Ketamine as a prophylactic against stress-induced depressive-like behavior. Biol Psychiat.

[30] Yang W, Xv M, Yang WC, Wang N, Zhang XZ, Li WZ (2015). Exogenous α-calcitonin gene-related peptide attenuates lipopolysaccharide-induced acute lung injury in rats. Mol Med Rep.

[31] Vago JP, Tavares LP, Riccardi C, Teixeira MM, Sousa LP (2021). Exploiting the pro-resolving actions of glucocorticoid-induced proteins Annexin A1 and GILZ in infectious diseases. Biomed Pharmacother.

[32] Dixon SJ, Lemberg KM, Lamprecht MR, Skouta R, Zaitsev EM, Gleason CE (2012). Ferroptosis: An iron-dependent form of nonapoptotic cell death. Cell.

[33] Herridge MS, Chu LM, Matte A, Tomlinson G, Chan L, Thomas C (2016). The RECOVER program: disability risk groups and 1-year outcome after 7 or more days of mechanical ventilation. Am J Respir Crit Care Med.

[34] Brimnes MK, Vangsted AJ, Knudsen LM, Gimsing P, Gang AO, Johnsen HE (2010). Increased level of both CD4+FOXP3+ regulatory T cells and CD14+HLA-DR^−^/low myeloid-derived suppressor cells and decreased level of dendritic cells in patients with multiple myeloma. Scand J Immunol.

[35] Raychoudhuri A, Shrivastava S, Steele R, Dash S, Ray RB (2010). Hepatitis C virus infection impairs IRF-7 translocation and Alpha interferon synthesis in immortalized human hepatocytes. J Virol.

[36] Wu M, Skaug B, Bi X, Mills T, Salazar G, Zhou X (2019). Interferon regulatory factor 7 (IRF7) represents a link between inflammation and fibrosis in the pathogenesis of systemic sclerosis. Ann Rheum Dis.

