# Supplementary material for: Irf7 regulates the expression of Srg3 and ferroptosis axis aggravated sepsis-induced acute lung injury
Source: Cell Mol Biol Lett. 2023 Nov 9;28:91. doi: 10.1186/s11658-023-00495-0 (PMC10634032; doi:10.1186/s11658-023-00495-0)
Supplement: Supplementary file 1 — Additional File 1: Fig. S1. Specific inhibition of the NF-κB signaling pathway or IRF7 weakens sepsis-induced lung injury. A Sepsis rats were treated with the NF-κB specific inhibitor JSH-23 or the IRF7 specific inhibitor AMG-232 via gavage. B, C qRT–PCR was used to detect changes in the expression levels of SRG3 in lung tissues of rats treated with JSH-23 or AMG-232. D H&E staining was used to examine the pathological structure of lung tissues in rats. E PAS staining was used to observe the proportion of glycogen in lung tissues of rats. F Flow cytometry was used to analyze the number of immune cells in the bronchoalveolar lavage fluid (BALF) of rats. G ELISA was used to detect the levels of inflammatory factors in the BALF of rats. H EB staining was used to observe vascular permeability in lung tissues of rats. I IHC staining was used to observe the staining intensity of SPC-1 in lung tissues of rats. J TUNEL staining was used to detect the proportion of apoptotic cells in lung tissues. Each experiment was repeated three times, and the data were presented in the form of mean plus or minus standard deviation. One-way ANOVA or two-way ANOVA was used for significance analysis between the data. After ANOVA, Tukey’s multiple comparison test was used for post hoc test. **P < 0.01, ***P < 0.001, ***P < 0.0001. Fig. S2. phos-p65 transcriptional activated Cox-2. A Verification of the binding relationship between phos-p65 and the Cox-2 promoter using ChIP–qPCR experiments. B, C Construction of a luciferase reporter vector containing the Cox-2 promoter sequence and transfection into HEK293T cells along with an DMSO or phos-p65 antagonists maslinic acid to measure luciferase activity. **P < 0.01, ***P < 0.001, ***P < 0.0001. [file 11658_2023_495_MOESM1_ESM.docx]

**
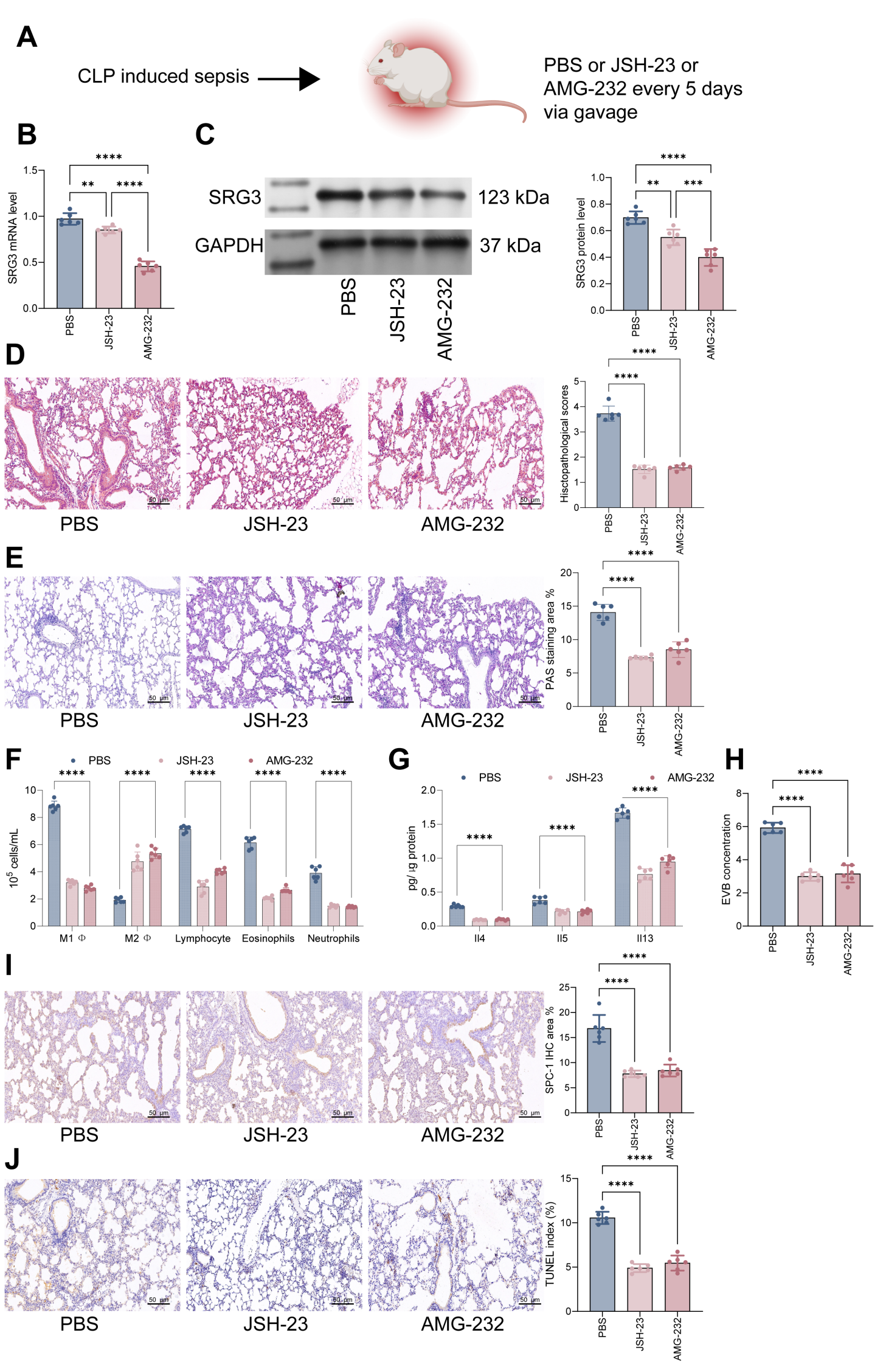
**

**Fig S1. Specific inhibition of the NF-kappaB signaling pathway or IRF7 weakens sepsis-induced lung injury.** A, sepsis rats were treated with the NF-kappaB specific inhibitor JSH-23 or the IRF7 specific inhibitor AMG-232 via gavage. B~C, qRT-PCR was used to detect changes in the expression levels of SRG3 in lung tissues of rats treated with JSH-23 or AMG-232. D, HE staining was used to examine the pathological structure of lung tissues in rats. E, PAS staining was used to observe the proportion of glycogen in lung tissues of rats. F, flow cytometry was used to analyze the number of immune cells in the bronchoalveolar lavage fluid (BALF) of rats. G, ELISA was used to detect the levels of inflammatory factors in the BALF of rats. H, EB staining was used to observe vascular permeability in lung tissues of rats. I, IHC staining was used to observe the staining intensity of SPC-1 in lung tissues of rats. J, TUNEL staining was used to detect the proportion of apoptotic cells in lung tissues.Each experiment was repeated three times, and the data were presented in the form of mean plus or minus standard deviation. 1-way ANOVA or 2-way ANOVA was used for significance analysis between the data. After ANOVA, Tukey's multiple comparison test was used for post hoc test. **P < 0.01, ***P < 0.001, ***P < 0.0001

**
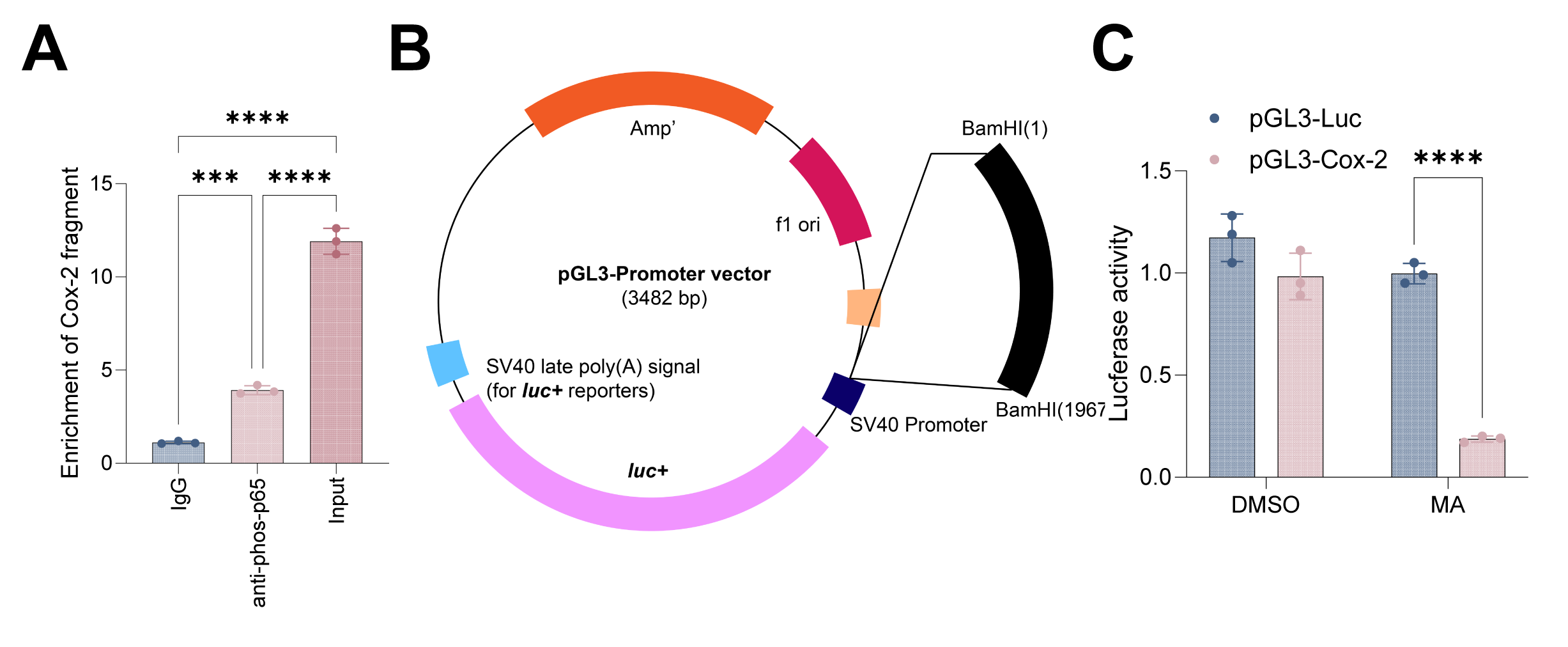
**

**Fig S2. phos-p65 transcriptional activated Cox-2.** A, Verification of the binding relationship between phos-p65 and the Cox-2 promoter using ChIP-qPCR experiments. B~C, Construction of a luciferase reporter vector containing the Cox-2promoter sequence and transfection into HEK293T cells along with an DMSO or phos-p65 antagonists Maslinic acid to measure luciferase activity. **P < 0.01, ***P < 0.001, ***P < 0.0001
